# Anti-Angiogenic Effect of Orally Available Pemetrexed for Metronomic Chemotherapy

**DOI:** 10.3390/pharmaceutics11070332

**Published:** 2019-07-13

**Authors:** Ruby Maharjan, Rudra Pangeni, Saurav Kumar Jha, Jeong Uk Choi, Kwan-Young Chang, Young Kweon Choi, Jin Woo Park, Youngro Byun

**Affiliations:** 1 Research Institute of Pharmaceutical Sciences, College of Pharmacy, Seoul National University, Seoul 08826, Korea; 2 Department of Pharmacy, College of Pharmacy and Natural Medicine Research Institute, Mokpo National University, Muan-gun, Jeonnam 58554, Korea; 3 Icure B&P, Global R&D center, Seoul 06649, Korea; 4 Department of Molecular Medicine and Biopharmaceutical Science, Graduate School of Convergence Science and Technology, College of Pharmacy, Seoul National University, Seoul 08826, Korea

**Keywords:** metronomic chemotherapy, anti-angiogenesis, oral delivery, low-dose therapy, pemetrexed

## Abstract

Metronomic chemotherapy (MCT) is defined as the frequent administration of low-dose chemotherapeutics, without long drug-free periods, with the exertion of antitumor activity exclusively through anti-angiogenic mechanisms. In this study, we have developed an orally available formulation of pemetrexed (PMX) for MCT. PMX was first complexed ionically with *N*^α^-deoxycholyl-l-lysyl-methylester (DCK) as the permeation enhancer. This was followed by dispersion with poloxamer 188 and Labrasol to form the solid oral formulation of PMX (PMX/DCK-OP). PMX/DCK-OP exhibited a 10.6-fold increase in permeability across a Caco-2 cell monolayer compared to PMX alone. This resulted in a 70-fold increase in the oral bioavailability of PMX/DCK-OP in mice over oral PMX alone. In the A549 xenograft model, tumor volume was reduced by 51.1% in the PMX/DCK-OP treated group compared to only 32.8% in the maximum tolerated dose (MTD)-treated group. Furthermore, PMX/DCK-OP exhibited a significant anti-angiogenic effect on the A549 xenograft mice when compared to the MTD-treated group, as indicated by microvessel density quantification for CD-31. In addition, PMX/DCK-OP enhanced the release of an endogenous angiogenesis inhibitor, thrombospondin-1 (TSP-1), into both the blood circulation and the tumor microenvironment. Therefore, due to its oral route of administration, PMX/DCK-OP appears to be a better alternative to the conventional treatment of PMX.

## 1. Introduction

There is a long history of using conventional chemotherapy for cancer treatment. Conventional therapy is administered at, or close to, the maximum tolerated dose (MTD), which kills both the tumor cells and rapidly growing normal cells [1]. These high doses also cause many adverse effects. To overcome them, a resting period of generally 2–3 weeks between doses is required. Consequently, this drug-free period allows tumor cells to regrow as they mobilize the circulating endothelial progenitor cells to elicit tumor neovascularization [2,3,4]. Recently, metronomic chemotherapy (MCT) has become an interesting alternative to conventional chemotherapy. MCT is defined as the frequent administration (daily, several times per week, or weekly) of low-dose chemotherapeutics without long drug-free (i.e., resting) periods [2,5,6]. Drug concentration in plasma remains constant, which continuously exposes the slowly proliferating tumor endothelial cells to the chemotherapeutic agent and prevents them from repairing and recovering. MCT attacks mainly proliferating endothelial cells rather than tumor cells, thereby preventing vessel formation and exploiting the anti-angiogenesis properties of the cytotoxic drug [3].

Initially, MCT was thought to exert its antitumor activity exclusively through anti-angiogenic mechanisms. However, extensive studies have uncovered additional mechanisms of action that include immunomodulation, induction of tumor dormancy, and acting directly through anti-cancer effects [2,5,6,7]. Thus, MCT is now considered to be multi-targeted chemotherapy. According to the original hypothesis proposed by Folkman, the anti-angiogenic paradigm of MCT relies greatly on targeting rapidly dividing tumor endothelial cells [2]. By targeting rapidly dividing tumor endothelial cells, MCT might be able to indirectly target both drug-sensitive and -resistant cancer cells by destroying existing vessels and preventing neoangiogenesis, hence leading to hypoxia and nutrient deprivation [1,2]. Moreover, MCT helps to release the potent endogenous angiogenesis inhibitor, thrombospondin-1 (TSP-1), which acts by binding to CD-36, expressed by activated endothelial cells [6,8,9,10,11]. TSP-1 can also inhibit the binding of vascular endothelial growth factor (VEGF) to its receptors, thus inhibiting tumor angiogenesis [11,12,13]. Bocci et al. confirmed that the anti-angiogenic and antitumor effects of low-dose continuous cyclophosphamide were lost in TSP-1-null C57BL/6 mice, whereas these effects were retained using an MTD schedule of the same drug. They concluded that TSP-1 is a secondary mediator of the anti-angiogenic effects observed during low-dose MCT regimens [9].

Many preclinical and clinical studies have been conducted using MCT. Browder et al. reported that a metronomic schedule of cyclophosphamide led to a significant antitumor effect in drug-resistant Lewis lung carcinoma xenografts, a result that was not possible using an MTD schedule [14]. Colleoni et al. also studied the effect of continuous treatment of low-dose cyclophosphamide and methotrexate in patients with metastatic breast cancer. They found that low doses of cyclophosphamide and methotrexate exhibited minimal toxicity and inhibited angiogenesis more effectively than the administered doses of MTD. Therefore, they proposed that an alternative hypothesis to that of direct toxicity on tumor cells must be responsible for the effects [15]. Similarly, MCT has demonstrated a better anti-cancer effect and significantly less toxicity compared with MTD in clinical studies of patients with breast, ovarian, prostate, and various other cancers [5,16,17,18].

Pemetrexed (LY-231514; *N*-[4-[2-(2-amino-3,4-dihydro-4-oxo-7H-pyrrolo[2,3-d]pyrimidin-5-yl)ethyl]benzoyl L-glutamic acid; PMX) is a multi-targeted, novel antifolate cytotoxic agent. Members of this class of drugs act by interfering with the functions of important biosynthetic enzymes, such as thymidylate synthase (TS), dihydrofolate reductase (DHFR), glycinamide ribonucleotide formyltransferase (GARFT), and aminoimidazole carboxamide formyltransferase (AICARFT). As these enzymes are involved in purine and pyrimidine synthesis, interfering with their function results in the inhibition of DNA and RNA synthesis [19,20,21]. The US Food and Drug Administration (FDA) has approved PMX in combination with cisplatin for the treatment of malignant pleural mesothelioma as well as advanced and metastatic non-small cell lung cancer (NSCLC). PMX alone is approved for maintenance treatment in patients with advanced NSCLC. In addition, a phase three clinical study reported that maintenance therapy with PMX is an effective and well-tolerated treatment option for patients with advanced non-squamous NSCLC. In that study, progression-free survival was 1.5 times higher in the PMX-treated group compared with the placebo group [20]. PMX and platinum-based therapies have been studied in combination with many immunotherapeutic drugs in various types of cancer. A phase 2 trial of PMX plus carboplatin with or without pembrolizumab showed significantly better rates of response and longer progression-free survival with the addition of pembrolizumab than with chemotherapy alone [22]. The successful study of pembrolizumab plus platinum-based doublet chemotherapy resulted in the FDA approval of this combination as first-line treatment in patients with metastatic non-squamous NSCLC.

MCT is generally administered daily or weekly in low doses [12]. Given the frequency required, an oral route of administration (possibly from home) is the most suitable option. However, the oral bioavailability of hydrophilic drugs is very low, possibly due to poor intestinal permeability. As such, various studies have examined strategies to enhance the oral absorption of hydrophilic drugs, including chemical modifications, complex formation with absorption enhancers, use of novel excipients, and coadministration of functional excipients [23,24,25]. Similarly, PMX should be administered intravenously as it exhibits low oral bioavailability, possibly due to its poor intestinal permeability [26]. To improve the oral absorption of PMX, Soni et al. prepared lipid-drug conjugated nanoparticles of PMX with Capryol 90 oil and Tween 80 and Labrasol surfactants in the ratio of 1:2:1. The lipid-drug conjugated nanoparticles were prepared by methods of solvent evaporation followed by cold homogenization. These nanoparticles demonstrated increased gut permeability and improved oral absorption, possibly due to the enhanced lipophilicity of PMX [27]. However, oral administration of lipid-based nanoparticles has several limitations including limited drug loading capacity of hydrophilic drugs, drug expulsion during storage, and particle size growth during storage [28]. Recently, we prepared an ion-pairing complex of PMX known as HP-β-CD/PMX/DCK/P188 by using a bile acid-derived permeation enhancer, *N*^α^-deoxycholyl-l-lysyl-methylester (DCK), in combination with two dispersants, 2-hydroxypropyl-β-cyclodextrin (HP-β-CD) and poloxamer 188 (P188). Then, we loaded the drug complex into the inner aqueous phase of multiple water-in-oil-in-water nanoemulsions, which resulted in a significant increase in oral bioavailability. However, the optimal formulation showed long-term instability during storage, which might be due to the incorporation of the drug complex in the inner aqueous phase of multiple nanoemulsions [29]. Hence, we designed an oral solid powder formulation of PMX with enhanced long-term stability.

The purpose of this study was to prepare an oral formulation of PMX for MCT and exploit its anti-angiogenic properties. In both in vitro and in vivo experiments, we studied anti-angiogenic effects of PMX including tubular formation, endothelial cell migration, Matrigel plug assays, etc. To enhance the intestinal membrane permeability and stability of PMX, a solid oral formulation of PMX (PMX/DCK-OP) was prepared using the ion-pairing complex of PMX and DCK in combination with two dispersing agents, P188 and Labrasol. In addition, enhanced absorption of PMX/DCK-OP across intestinal membranes was expected due to the increased lipophilicity caused by DCK, interaction with bile acid transporters, the stable formation of micelles in contact with the aqueous phase, dispersant activity of P188, and opening of tight junctions by Labrasol. Therefore, to determine the potential of the powder formulation to enhance oral absorption, we used an artificial membrane and Caco-2 cell monolayer to study the permeability of PMX/DCK and PMX/DCK-OP. In addition, the effect of PMX/DCK-OP at different doses on oral bioavailability and tumor growth was evaluated in vivo. Finally, we studied the anti-angiogenic and antitumor effects of the optimized metronomic dose of PMX/DCK-OP and MTD dose of PMX. In particular, we compared the effects of these two treatments on tumor inhibition, blood vessel formation in a Matrigel plug, the expression of microvessels in tumor tissue, and the level of TSP-1 expression in circulating blood and tumor tissue.

## 2. Materials and Methods

### 2.1. Materials

PMX disodium hemipentahydrate was purchased from Shipla Medicare (Karnataka, India). Caprylocaproyl macrogol-8-glycerides (Labrasol) were provided by Gattefossé (Saint Priest, France). Deoxycholic acid (DOCA), Drabkin’s reagent, and Duolink in situ mounting medium with DAPI were obtained from Sigma-Aldrich (St. Louis, MO, USA). Polyoxyethylene (160) polyoxypropylene (30) glycol (poloxamer 188; P188) was obtained from BASF (Ludwigshafen, Germany). Matrigel membrane matrix (growth factor reduced) was purchased from Corning (Manassas, VA, USA). VEGF_165_ and basic fibroblast growth factor (bFGF) were purchased from Pepro Tech (Rocky Hill, NJ, USA). Brij-35 solution was obtained from Thermo Fisher Scientific (Waltham, MA, USA). The solvents used in high-performance liquid chromatography (HPLC) were obtained from Merck and Thermo Fisher Scientific (Waltham, MA, USA). Polyethylene glycol-conjugated rat anti-mouse CD-31 antibody was purchased from BD Biosciences (Franklin Lakes, NJ, USA). Anti-thrombospondin-1 antibody and Goat anti-rabbit Alexa Fluor 488 were purchased from Abcam (Cambridge, UK) and Invitrogen (Carlsbad, CA, USA), respectively.

### 2.2. Animals

C3H/Ne mice (male, 6-week-old), C57BL/6 mice (female, 6-week-old), and BALB/c nude mice (female, 6-week-old) were purchased from Orientbio (Gyeonggi-do, Republic of Korea). The animals were housed under standard housing conditions of temperature (23 °C ± 2 °C), relative humidity (55% ± 10%), and light (12/12-h light/dark cycle). The animals had ad libitum access to a standard laboratory diet (Nestlé Purina, St Louis, MO, USA) and ion-sterilized tap water. Ethical approval for this study was obtained from the Institutional Animal Care and Use Committee (IACUC) of Seoul National University (Seoul, Republic of Korea) on August 1, 2018 (approval No. SNU-170421-3-5). All animal experiments were performed in accordance with the National Institutes of Health Guidelines for the Care and Use of Laboratory Animals and the guidelines of the IACUC of Seoul National University.

### 2.3. In Vitro Anti-Angiogenic Effect of PMX

#### 2.3.1. Cytotoxicity Assay

The effects of conventional and metronomic administration of PMX were assessed by cell cytotoxicity assays. Adenocarcinomic human alveolar basal epithelial cells (A549 cells) were purchased from the American Type Culture Collection (ATCC, Manassas, VA, USA) and cultured in Dulbecco’s modification of Eagle medium (DMEM; Corning, Manassas, VA, USA) supplemented with 10% fetal bovine serum (FBS; Corning, Manassas, VA, USA) and 1% penicillin/streptomycin (Gibco, Thermo Fisher Scientific). Human umbilical vein endothelial cells (HUVECs) were purchased from PromoCell (Heidelberg, Germany) and were cultured in Endothelial Cell Growth Medium MV2 (ECGM, PromoCell, Heidelberg, Germany) supplemented with 10% FBS and 1% penicillin/streptomycin. A549 cells and HUVECs were seeded at the concentration of 5 × 10^3^ cells per well on 96-well culture plates and allowed to grow for 24 h. The cells were then treated with PMX at different concentrations of 0, 0.01, 0.1, 1, and 10 µg/mL and incubated for 72 h in 5% CO_2_ at 37 °C. Metronomic dosing was simulated by aspirating and replacing the media with freshly prepared media containing PMX every 24 h [12,30]. Then, a cytotoxicity assay was performed using the Cell Counting Kit-8 (CCK-8; Dojindo Molecular Technologies, Rockville, MD, USA) according to the manufacturer’s instructions. Briefly, after the completion of treatment, 10 µL of 2-(2-methoxy-4-nitrophenyl)-3-(4-nitrophenyl)-5-(2,4-disulfophenyl)-2H-tetrazolium monosodium salt (WST-8) reagent was added to each well and incubated at 37 °C for 2 h. Absorbance was then measured in the microplate reader (Synergy HTX; Bio-Tek, Winooski, VT, USA) at 450 nm.

#### 2.3.2. Tube Formation Assay

The in vitro endothelial tube formation assay was performed as described in our previous studies [31,32]. Briefly, 100 µL Matrigel were loaded into each well of a 96-well culture plate and incubated at 37 °C for 30 min, for polymerization. Then, 3 × 10^4^ HUVECs in ECGM media with 10% FBS and PMX at different concentrations (0, 0.1, 1, and 10 µg/mL) were loaded over Matrigel into each well. After 24-h incubation at 37 °C, the number of branches and nodes were counted at 20× magnification using a microscope (Eclipse Ts2, Nikon Instruments Inc., Melville, NY, USA). The acquired images were analyzed with ImageJ for quantification.

#### 2.3.3. Scratch Wound Recovery Assay

A wound was caused by scratching to remove a monolayer of the HUVECs. Then, cell migration within the wound was assessed by a wound healing assay [33]. Wound healing was performed by seeding 5 × 10^5^ HUVECs per well in a 24-well plate. The plate was incubated in 5% CO_2_ at 37 °C until the cells reached full confluency. After confluence was attained, a straight-line scratch was made by using a 200 µL pipette tip. The plate was then washed a few times to remove the detached cells. An image of the scratch wound was captured at this time point (0 h). Then, the cells were incubated in 5% CO_2_ at 37 °C after treatment with various concentrations of PMX (0, 0.1, 1, and 10 µg/mL). After incubating for 8 h, the cells were observed under the microscope for wound recovery and scratch confluency. The acquired images were quantified using ImageJ software.

### 2.4. Preparation and Characterization of PMX/DCK-OP

In the present study, DCK was used to enhance oral permeation and was synthesized by conjugation of positively charged lysine to DOCA, as previously described [19]. Next, an ionic complex of PMX with DCK (PMX/DCK) was prepared by dissolving PMX (50 mg) in 10 mL of deionized water. DCK (66.8 mg) was separately dissolved in 10 mL deionized water and added dropwise to the PMX solution, with continuous stirring, at a molar ratio of 1:1. The PMX/DCK solution was freeze-dried at −70 °C to obtain a PMX/DCK powder. To improve oral absorption of PMX/DCK, an oral PMX powder was prepared by ion-pairing complexation of PMX and DCK in the presence of two dispersing agents, Labrasol and P188. In brief, 50 mg PMX was dissolved in 10 mL P188 solution (50 mg/mL in deionized water) containing 375 µL Labrasol. Separately, 66.8 mg DCK was dissolved in 8 mL deionized water and the solution was added dropwise to the PMX solution with vortex mixing; the molar ratio was 1:1. Next, 80 µL 1 M NaOH was added to ensure that the pH was pH 7. Finally, the PMX/DCK solution containing P188 and Labrasol was freeze-dried at −70 °C, yielding a solid powder termed PMX/DCK-OP.

To confirm complexation between PMX and DCK and maintenance of such complexation in PMX/DCK-OP as well as the crystalline properties of PMX, DCK, P188, and PMX/DCK, a physical mixture of PMX, DCK, P188, and Labrasol, and separately PMX/DCK-OP were assessed via powder X-ray diffraction (PXRD). Powder samples were placed on an adhesive support (0.5 mm in thickness) and analyzed with the aid of a D8 Advance diffractometer (Bruker AXS GmbH, Karlsruhe, Germany) operating at 40 mA and 40 kV to deliver copper (Cu-Kα1) radiation (λ = 1.5418 Å) in the step-scan mode over a diffraction angle (2θ) of 5–50° at a scan rate of 0.02°/s. We also measured the particle size, the polydispersity index (PDI), and the zeta potential of PMX/DCK-OP in water, to confirm micelle formation in the solution state. PMX/DCK-OP was diluted with deionized water (1:20, *w*/*v*), followed by sonication for 1 min to minimize scattering. Next, particle size and surface charge were measured using a dynamic laser light scattering instrument (Malvern Zetasizer Nano ZS90; Malvern Instruments, Malvern, UK) operating at 25 °C. The particle size and morphology of PMX/DCK-OP were confirmed via transmission electron microscopy (TEM). PMX/DCK-OP dispersed in deionized water (1:100, *w*/*v*) was negatively stained with 2% (*w*/*v*) phosphotungstic acid, dropped onto a copper grid, and examined via high-resolution TEM (HR-TEM; JEM-200; JEOL, Tokyo, Japan).

### 2.5. In Vitro Artificial Intestinal Membrane and Caco-2 Cell Monolayer Permeability

A parallel artificial membrane permeability assay (PAMPA, BD Biosciences, San Jose, CA, USA) was performed to measure the in vitro intestinal membrane permeability of PMX, PMX/DCK, and PMX/DCK-OP as described previously [34]. In brief, each sample was diluted with phosphate-buffered saline (PBS, pH 6.8) to a final concentration of 200 µg/mL of PMX. Next, 200 µL of the sample solution were added to each well of the donor compartment. Similarly, each well of the acceptor compartment was filled with 300 µL of PBS (pH 6.8) and coupled with the donor compartment. The plate assembly was detached after 5-h incubation at room temperature and samples were withdrawn from both the acceptor and donor compartments. The concentration of PMX that permeated through the artificial intestinal membrane was measured using HPLC with a C18 column (4.6 × 250 mm, 5 µm, 100 Å) at 25 °C. Each sample collected from the acceptor and donor compartments was injected (20 µL-injection volume) into the HPLC system, with the mobile phase consisting of water (pH 3.5 adjusted with phosphoric acid)–acetonitrile (80:20, *v*/*v*) and run at a flow rate of 1 mL/min. The quantification of PMX or PMX/DCK was carried out at 254 nm. The effective permeability (*P_e_*) of each drug was calculated using the following formula: *P_e_* = −*ln*(1 − C_A_[*t*]/C_equilibrium_)/(A × [1/V_D_ + 1/V_A_] × *t*), where *P_e_* is the permeability (cm/s), A is the effective surface area of the filter (0.228 cm^2^), V_D_ is the donor well volume (0.2 mL), V_A_ is the receptor well volume (0.3 mL), *t* is the total incubation time in seconds, C_A_(*t*) is the concentration of drug in the receptor well at time *t*, and C_equilibrium_ represents (C_D_[*t*] × V_D_ + C_A_[*t*] × V_A_)/(V_D_ + V_A_), where C_D_(*t*) denotes the concentration of drug in the donor well at time, *t*.

Similarly, the permeability of PMX, PMX/DCK, and PMX/DCK-OP across a Caco-2 cell monolayer was also measured. Caco-2 cells (ATCC^®^ HTB-37^TM^) at a density of 1 × 10^5^ cells/well were seeded into each 24-well Transwell^®^ filter insert (pore size 0.4 µm, surface area 0.33 cm^2^, Corning Incorporated, Corning, NY, USA) in complete Dulbecco’s Modified Eagle Medium (DMEM) supplemented with 10% FBS and 1% penicillin/streptomycin. The culture medium was changed every second day and allowed to grow and differentiate for 14–16 days. The cellular monolayers with a transepithelial electrical resistance (TEER) greater than 350 Ω·cm^2^ were used for the transport experiments. The apical and basolateral compartments were stabilized for 20 min with 0.1 and 0.6 mL of preheated (37 °C) Hanks’ balanced salt solution (HBSS), respectively. Then, solution in the apical compartment was replaced with 100 µL of PMX, PMX/DCK, or PMX/DCK-OP diluted with HBSS (equivalent to 100 µg/mL PMX), and the basolateral compartment was replaced with 0.6 mL of fresh HBSS. The plate assembly was incubated at 37 °C, and 100 µL of the PMX or PMX/DCK sample that permeated through the Caco-2 cell monolayer was withdrawn at 0.5, 1, 2, 3, 4, and 5 h from each basolateral compartment and replaced with 100 µL of fresh HBSS. The amount of PMX or PMX/DCK that permeated through the Caco-2 monolayer was determined by HPLC with a UV detector, as described above. The apparent permeability coefficient (*P_app_*) of PMX, PMX/DCK, or PMX/DCK-OP was calculated according to the following equation: *P_app_* = d*Q*/d*t* × 1/(*S* × *C*_0_), where d*Q*/d*t* indicates the linear appearance rate of mass in the basolateral compartment (μmoL/s), *C*_0_ is the initial concentration of PMX, PMX/DCK, or PMX/DCK-OP in the apical compartment (μmoL/mL), and *S* is the surface area of the monolayer (cm^2^).

### 2.6. Oral Bioavailability of PMX/DCK-OP in Mice

Male C3H/Ne mice (20–25 g) were used for the pharmacokinetic study and equilibrated in optimal room conditions. The mice were fasted overnight and their gastric pH was neutralized with sodium bicarbonate solution (3% [*v*/*v*]) prior to the study. The animals were treated with 100 µL of PMX solution (20 mg/kg) by intravenous administration or 200 µL of PMX (50 mg/kg), PMX/DCK-OP (10 mg/kg PMX), PMX/DCK-OP (20 mg/kg PMX), and PMX/DCK-OP (40 mg/kg PMX) by oral administration. The blood samples were collected from the retro-orbital plexus using capillary tubes at predetermined time points and stabilized immediately by mixing with sodium citrate solution (3.8% [*w*/*v*]). The plasma was isolated by centrifuging at 2000× *g* for 15 min at 4 °C and then stored at −70 °C until further analysis.

The plasma concentration of PMX was determined using liquid chromatography/mass spectrometry (LC/MS), as described previously [29]. In brief, the plasma samples were defrosted and centrifuged at 2500× *g* for 5 min at 4 °C and mixed with 300 µL of 2% NH_4_OH in water along with 100 µL of standard solution or plasma sample and 10 µL of 4-[*N*-(2,4-diamino-6-pterinidinylmethyl)-*N*-methylamino]benzoic acid hemihydrochloride hydrate (DAMPA; 5 µg/mL, IS). After vortex mixing for 2 min, the entire mixture was subjected to solid-phase extraction using a Plexa Bond Elut PAX cartridge (30 mg, 1 mL; Agilent Technologies, Santa Clara, CA, USA) as per the manufacturer’s instructions. In short, before loading the standard or plasma sample mixture into the cartridge, conditioning of the cartridge with each 500 µL of methanol and deionized water was performed. The impurities, as well as unadsorbed samples in the cartridge, were washed with deionized water followed by methanol (500 µL each). Then, the adsorbed samples were eluted using 2 × 250 µL of 5% formic acid prepared in methanol and further dried using a centrifugal evaporator (Genevac Ltd., Ipswich, Suffolk, UK). After the dried sample residue was reconstituted with 100 µL of 5% formic acid prepared in methanol, the concentration of PMX in plasma was determined using an Agilent 6120 Quadruple LC/MS system connected to a Phenomenex Luna C18 column (100 × 2 mm, 3 µm) at room temperature. Acetonitrile–0.34% formic acid solution (15:85, *v*/*v*) was used as the mobile phase, which was delivered at a flow rate of 0.2 mL/min. The ionization of PMX and IS were performed using an Electrospray Ionization (ESI) source in positive ion mode with selected ion monitoring (SIM). The LC/MS interface was set at the following conditions: capillary voltage at 3.5 kV; drying gas flow rate at 3.1 L/min; and drying gas temperature at 300 °C. At this working voltage, a quantitative analysis of the protonated molecular ions was performed at ([M + H]^+^ = 428) and ([M + H]^+^ = 326.1) for PMX and DAMPA, respectively.

### 2.7. In Vivo Matrigel Plug Assay of PMX/DCK-OP

A Matrigel plug assay was used to evaluate the in vivo anti-angiogenic activities of PMX/DCK-OP, as previously described with some modifications [9,31]. A total of 500 µL of Matrigel (Corning, Manassas, VA, USA) supplemented with or without 500 ng/mL of VEGF_165_ and 500 ng/mL of bFGF was injected subcutaneously into the ventral side of each 6-week-old female C57BL/6 mouse. The mice inoculated with Matrigel only (without any supplement) were used as the negative control group, whereas those treated with both Matrigel and supplements were used as the positive control and treatment groups. The treatment groups were distributed into four groups as follows: PMX/DCK-OP (5 mg/kg PMX), PMX/DCK-OP (10 mg/kg PMX), PMX/DCK-OP (20 mg/kg PMX), and PMX/DCK-OP (40 mg/kg PMX), and received once-a-day oral PMX/DCK-OP equivalent to 5 mg/kg, 10 mg/kg, 20 mg/kg, and 40 mg/kg of PMX, respectively. No treatment was administered to the negative and positive control groups. Seven days after administration, all mice were sacrificed and the Matrigel plugs were extracted. Hemoglobin content was analyzed immediately by homogenizing each plug in 200 µL hypotonic lysis buffer (0.1% Brij-35 solution) and centrifuging for 20 min at 15,000 rpm. Twenty µL of the supernatant were incubated with 100 µL Drabkin’s reagent in a 96-well plate and incubated for 15 min at room temperature. Absorbance was measured at 540 nm using a microplate reader.

### 2.8. In Vivo Antitumor Efficacy of PMX/DCK-OP in Mice

To evaluate the antitumor efficacy of PMX/DCK-OP, A549 cells (1 × 10^7^ cells/mouse) were subcutaneously inoculated into the right dorsal flanks of 6-week-old female BALB/c nude mice. When the tumor volume reached 50–70 mm^3^, the mice were randomly divided into the following groups (10 mice/group): (1) Control; (2) PMX/DCK-OP (10 mg/kg PMX); (3) PMX/DCK-OP (20 mg/kg PMX); and (4) PMX/DCK-OP (40 mg/kg PMX). The mice received once-daily normal saline or PMX/DCK-OP at 10, 20, or 40 mg/kg orally. Tumor length and width were measured using a caliper every 3 days, and tumor volume was calculated as: length × (width)^2^ × 0.5, where the length was the largest tumor diameter. The study ran for 24 days in total.

### 2.9. In Vivo Anti-Angiogenic Effect of Oral Metronomic Chemotherapy of PMX-DCK-OP

#### 2.9.1. In Vivo Anti-Angiogenic Effect

To compare anti-angiogenic activities between the MTD dose of PMX and oral MCT of PMX/DCK-OP, 500 µL of Matrigel incorporating 500 ng/mL of VEGF_165_ and 500 ng/mL of bFGF were subcutaneously injected into the ventral side of each 6-week-old female C57BL/6 mouse. The mice were then randomly assigned to four groups of five mice each as follows: Negative and Positive control (without drug administration), PMX-MTD (once-a-week intraperitoneal injection of 300 mg/kg PMX), and PMX/DCK-OP (once-daily oral administration of PMX/DCK-OP at 20 mg/kg PMX). In addition, the mice inoculated with Matrigel alone were grouped as a negative control. At 7 days after treatment, all mice were sacrificed and Matrigel plugs were extracted. Then, the hemoglobin content was analyzed immediately as described in Section 2.7.

#### 2.9.2. In Vivo Antitumor Efficacy

To evaluate the antitumor efficacy of the PMX MTD dose and oral MCT dosing of PMX/DCK-OP, 6-week-old female BALB/c nude mice were subcutaneously injected with A549 cells (1 × 10^7^ cells/mouse) in the right dorsal flanks. After the tumor volume reached 50–70 mm^3^, the mice were randomly assigned to three groups of six mice each, as follows: Control (once-daily oral administration of normal saline); PMX-MTD (once-weekly intraperitoneal injection of 300 mg/kg PMX); and PMX/DCK-OP (once-daily oral administration of 20 mg/kg PMX). Tumor length and width were measured using a caliper every other day for 26 days, and tumor volume was calculated as: length × (width)^2^ × 0.5. On the last day of observation, blood samples were collected from the retro-orbital plexus into capillary tubes and immediately mixed with sodium citrate solution (3.8% [*w*/*v*]). Plasma was isolated by centrifugation at 2000× *g* for 15 min at 4 °C and stored at −70 °C prior to analysis. Isolated tumor tissues were fixed in 10% (*v*/*v*) formalin and embedded in paraffin prior to immunofluorescence staining.

#### 2.9.3. Circulating TSP-1 by Immunoassay

The expression level of circulating TSP-1 in the blood plasma collected at the end of the experiment was evaluated using the mouse TSP-1 ELISA kit (Elabscience, Huston, TX, USA) following the manufacturer’s instructions. Briefly, the standard working solution was added to the first two columns, as each concentration of the solution is added in duplicate to one well each, side by side (100 μL for each well). The samples were then added to the other wells (100 μL for each well). The plate was then covered with the sealer provided and incubated for 90 min at 37 °C. After incubation, the liquid was removed from each well without washing and 100 μL of working solution of Biotinylated Detection Ab was added immediately to each well. The plate was then covered with the sealer, mixed gently, and incubated for 1 h at 37 °C. After the solution was removed from each well, 350 μL of washing buffer was added to each well, followed by soaking for 2 min. Then, the solution was decanted from each well and patted dry using clean absorbent paper. This washing step was repeated three times. Next, 100 μL horseradish peroxidase (HRP) conjugate working solution was added to each well and incubated at 37 °C for 30 min after covering with the plate sealer. The solution was aspirated from the plate and the washing step was repeated five times as described above. After washing and drying, 90 μL of Substrate Reagent were added to each well and incubated for about 15 min at 37 °C by protecting the plate from light. Finally, 50 μL of Stop Solution were added to each well and the optical density of each well was taken at once with a microplate reader (Synergy HTX, Bio-Tek, Winooski, VT, USA) set to 450 nm.

#### 2.9.4. Immunofluorescence Study

At the end of our study, tumor tissues from mice inoculated with A549 cells were collected for histological analysis. Tumor tissues were fixed in 10% formalin and cut into 4-μm thin sections and blocked by paraffin. Paraffin-blocked tissue sections were deparaffinized by incubating in a hot-air oven at 65 °C for 15 min and then retrieved by immersing the slides in xylene and a series of alcohol solutions (100%, 90%, 80%, and 70%, *v*/*v*). After washing in PBS (1×), antigens were retrieved by immersing the slides in citrate buffer in a heated steamer. After washing with PBS and blocking, the respective antibody treatment was performed overnight. For the detection of microvessels, the tissue sections were stained with PE-conjugated rat anti-mouse CD-31 antibody (1:200) overnight and then embedded with DAPI mixed mounting media. For the detection of thrombospondin-1, the tissue sections were stained with anti-thrombospondin-1 antibody (1:100) overnight at 4 °C, washed again, stained with goat anti-rabbit Alexa Fluor 488 (1:200) for 1 h, and then embedded with DAPI mixed mounting media. The sections were visualized under confocal microscopy (LSM 710, Carl Zeiss, Germany).

### 2.10. Pharmacokinetic and Statistical Analyses

Pharmacokinetic parameters were determined using a non-compartmental method with WinNonlin^®^ software (ver. 5.3; Pharsight Corporation, Mountain View, CA, USA). GraphPad Prism (ver. 5, GraphPad Software, San Diego, CA, USA) was used for the statistical analysis. All data were evaluated using the Student’s *t*-test for comparisons between two mean values for unpaired data or one-way analysis of variance followed by Tukey’s multiple-comparisons test among three or more mean values for unpaired data and expressed as the mean ± standard error of the mean (SEM). In all analyses, *p* < 0.05 indicated statistical significance.

## 3. Results and Discussion

### 3.1. Cytotoxic Effects of Conventional and Metronomic Doses of PMX

**Figure 1:**
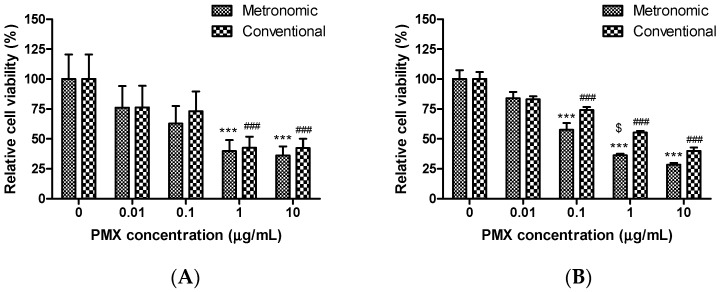
In vitro cytotoxic effects of metronomic and conventional doses of PMX on (**A**) A549 cells and (**B**) HUVECs. Cells were exposed to PMX at various concentrations (0–10 µg/mL) for 72 h. Metronomic dosing was simulated by aspirating and replacing the medium with fresh drug-containing medium after 24 and 48 h. Data are presented as means ± SEM (*n* = 8 for each group). *** *p* < 0.001 compared to the untreated control group (metronomic dosing). ^###^ *p* < 0.001 compared to the untreated control group (conventional dosing). ^$^ *p* < 0.05 compared to the conventional treatment group at the same drug concentration. A549, human lung carcinoma cell line; HUVEC: human umbilical vein endothelial cell line; PMX: pemetrexed.

In vitro simulations of the conventional and metronomic cytotoxic effects of PMX were performed in A549 cells and HUVECs using the CCK-8 assay. PMX was added to cells at various concentrations (0, 0.01, 0.1, 1, and 10 µg/mL) followed by incubation for 72 h. To mimic metronomic dosing, the drug-containing medium was changed every 24 h. A strong, concentration-dependent cytotoxic effect was evident when the cells were exposed to either conventional or metronomic PMX treatment (Figure 1). However, over the concentration range tested, the metronomic treatment groups exhibited greater cytotoxicity than the conventional treatment groups. PMX at 1 µg/mL decreased A549 cell viability by more than 50% when dosing was either metronomic or conventional; the residual cell viabilities were 39.8 ± 9.31% and 42.6 ± 9.18%, respectively (*p* < 0.001) compared to untreated control cells (Figure 1A). For HUVECs, PMX at 0.1 to 10 µg/mL was associated with a significant reduction in cell viability after both metronomic and conventional treatments, but with PMX treatment at 1 µg/mL, cell viability was 1.52-fold lower after metronomic than conventional treatment (*p* < 0.05); metronomic dosing was thus more effective against endothelial than tumor cells. Bocci et al. similarly found that metronomic dosing was more effective for reducing endothelial versus tumor cell viability [35]. For A549 cells, the half maximal inhibitory concentration (IC_50_) values of the metronomic and conventional treatments were 0.179 and 0.272 µg/mL, respectively. As the IC_50_ value was thus 1.52-fold lower in the metronomic group, it may be more potent than conventional treatment. The PMX IC_50_ values for HUVECs were 0.059 and 0.621 µg/mL (metronomic and conventional treatments, respectively). The IC_50_ value increased 10.5-fold in the metronomic group. Similar results have been observed in many in vitro experiments using topotecan, irinotecan, capecitabine, paclitaxel, docetaxel, vinorelbine, and ibandronate; metronomic treatment was more potent than conventional treatment [12,30,33,35,36]. Further, HUVECs are more sensitive than lung cancer cells to PMX metronomic treatment (IC_50_ values of 0.059 and 0.179 µg/mL, respectively). This may be because continuous in vitro exposure of endothelial cells to PMX significantly increases TSP-1 expression and secretion into the medium [35]. Thus, compared to conventional dosing, metronomic dosing is more potent, effectively enhancing the utility of PMX. Prolonged metronomic treatment was more effective than a short-term treatment resembling conventional chemotherapy.

### 3.2. Anti-Angiogenic Effect of PMX

**Figure 2:**
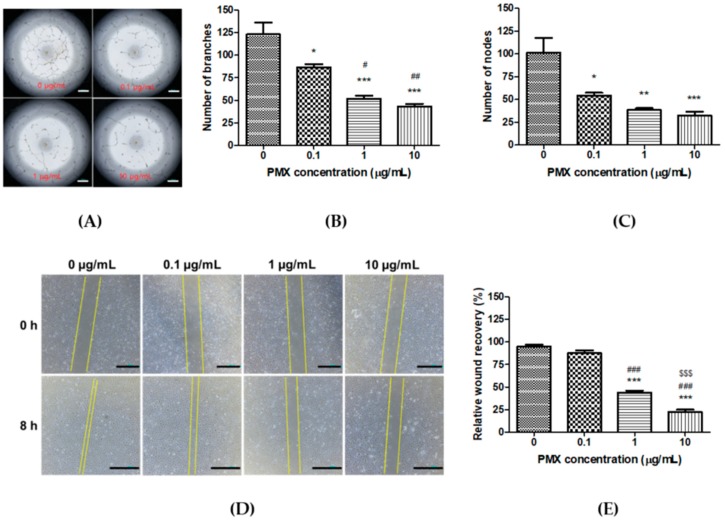
In vitro anti-angiogenic effect of PMX. (**A**) Dose-dependent effect of PMX on tube formation in HUVECs. Magnification 20×. Bar means 500 µm. (**B**) Quantitative image analysis of HUVEC tubes by ImageJ to determine the number of branches. (**C**) Quantitative image analysis of HUVEC tubes by ImageJ to determine the number of nodes formed. (**D**) Scratch wound recovery in HUVECs at 0 h and after 8 h of treatment with PMX at various concentrations (0, 0.1, 1, and 10 µg/mL). Magnification 40×. Bar means 1 mm. (**E**) Quantification of relative wound recovery. Data are presented as mean ± SEM (*n* = 5 for each group). * *p* < 0.05, ** *p* <0.01, *** *p* < 0.001 compared to the control. ^#^ *p* < 0.05, ^##^ *p* < 0.01, ^###^ *p* < 0.001 compared to 0.1 µg/mL PMX. ^$$$^ *p* < 0.001 compared to 1 µg/mL PMX.

Next, we conducted various experiments to confirm the anti-angiogenic effect of PMX. First, we performed a tube formation experiment by using different concentrations of PMX (0, 0.1, 1, and, 10 µg/mL) in HUVECs. Well-organized branches were present in the non-treated control group, while the numbers of tubes and tubular junctions were significantly reduced in the PMX-treated groups in a dose-dependent manner (Figure 2A). Tube formation was significantly decreased by 1.42-fold in the 0.1 µg/mL PMX (*p* < 0.05), 2.36-fold in the 1 µg/mL PMX (*p* < 0.01), and 2.84-fold in the 10 µg/mL PMX (*p* < 0.01) treatment groups compared with the control group (Figure 2B). Similarly, the number of nodes was reduced by 1.87-fold in the 0.1 µg/mL PMX (*p* < 0.05), 2.65-fold in the 1 µg/mL PMX (*p* < 0.05), and 3.12-fold in the 10 µg/mL PMX (*p* < 0.01) treatment groups compared with the control group (Figure 2C). The number of both tubes and nodes in HUVECs were effectively reduced by PMX in a dose-dependent manner. This indicates that PMX significantly inhibited the ability of HUVECs to form tubes by inhibiting the proliferation and migration of endothelial cells.

Next, we performed a scratch wound assay on HUVECs for further analysis of the anti-angiogenic effect of PMX. Eight hours later, wound recovery was observed under the microscope and images were captured (Figure 2D). The wound area almost fully recovered (95.0 ± 2.04%) in the non-treated group. On the other hand, a concentration-dependent inhibition of wound recovery was observed with 87.5 ± 3.22%, 43.8 ± 2.39%, and 22.5 ± 2.50% recovery measured at concentrations of 0.1 µg/mL PMX, 1 µg/mL PMX (*p* < 0.001), and 10 µg/mL PMX (*p* < 0.001), respectively, in the treated wells compared with the initial scratch (Figure 2E). Scratch wound recovery was significantly reduced in a dose-dependent manner by PMX.

The ability of PMX to inhibit tube formation and wound recovery verifies the anti-angiogenic property of PMX. Thus, PMX inhibits the ability of endothelial cells to proliferate and migrate. Based on these results, PMX can be considered a strong candidate for MCT.

### 3.3. Characterization of PMX/DCK-OP

**Figure 3:**
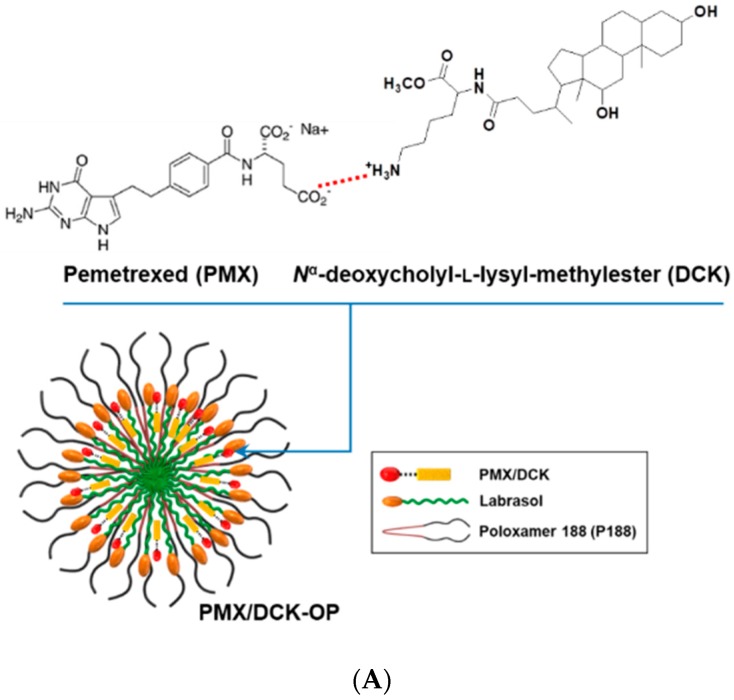

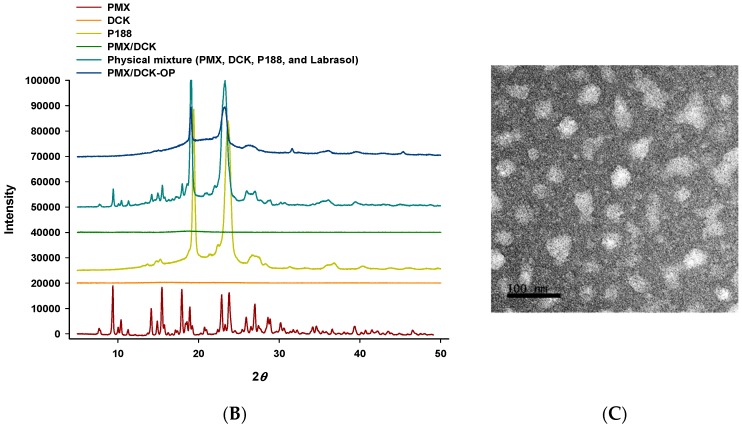
(**A**) A structural diagram of the ion-paired complex of PMX and *N*^α^-deoxycholyl-l-lysyl-methylester (DCK), and a schematic representation of PMX/DCK-OP micelles. (**B**) Powder X-ray diffractograms of PMX, DCK, P188, and PMX/DCK; a physical mixture of PMX, DCK, P188, and Labrasol; and PMX/DCK-OP. (**C**) Transmission electron micrograph of PMX/DCK-OP micelles. Scale bar: 100 nm.

We formulated an ion-paired complex of PMX and DCK to improve the intestinal membrane permeability and oral bioavailability of PMX. An optimal oral powder of PMX was prepared using a solid dispersion technique, employing Labrasol and P188 to increase PMX amphiphilicity; the drug spontaneously self-assembled into nanomicelles (Figure 3A). To confirm ion-pairing complexation of PMX and DCK in PMX/DCK-OP, the PXRD spectra of PMX, DCK, P188, and physical mixtures thereof were compared to those of PMX/DCK and PMX/DCK-OP (Figure 3B). Pure PMX exhibited crystalline peaks (2θ values) of 9.40, 10.40, 11.28, 14.14, 14.90, 15.48, 17.94, 18.94, 22.88, 23.82, 25.90, 26.52, 28.62, 29.66, and 30.16°. P188 also existed in a crystalline form; two peaks were observed at 19.41 and 23.68° over the 2θ range. The crystalline peaks of PMX and P188 were also evident in the physical mixture, indicating that both PMX and P188 remained in crystalline form. However, the X-ray diffractograms of the freeze-dried powders PMX/DCK and PMX/DCK-OP lacked the PMX peaks, suggesting that PMX had become molecularly dispersed with DCK and existed in an amorphous form after ion-pairing complexation. Furthermore, PMX/DCK was completely dispersed with P188 and Labrasol at the molecular level, resulting in formation of amorphous complexes. However, further studies including Fourier transform infrared (FT-IR) spectroscopy are required to identify the physicochemical interactions between PMX, DCK, P188, and Labrasol in the complexes.

Next, to confirm formation of self-assembled PMX/DCK-OP nanomicelles in aqueous solution, we measured the average particle size, PDI, and zeta potential of the micelles via dynamic laser light scattering. Prior to incorporating PMX or PMX/DCK into oral formulations, P188 and Labrasol formed loosely packed micelles; the size, PDI, and zeta potential were 402 ± 39.3 nm, 0.922 ± 0.070, and 0.048 ± 0.118 mV, respectively. DCK incorporation into micelles formed by P188 and Labrasol significantly changed the particle size, PDI, and zeta potential (104 ± 0.320 nm, 0.034 ± 0.021, and 40.6 ± 6.30 mV, respectively), indicating that positively charged DCK molecules were well oriented in the micelles. After dilution with deionized water, the size of the PMX/DCK-OP micelles was 130 ± 7.92 nm, as measured using a particle size analyzer. Such a nanosize facilitates effective intracellular uptake. The PDI was low (0.339 ± 0.055), confirming that the particle size distribution was uniform and suggesting the formation of stable micelles (Figure 3B). Further, the zeta potential of the diluted powder formulation was −7.57 ± 1.35 mV, suggesting that PMX formed ion-paired complexes with DCK. TEM of PMX/DCK-OP dispersed in deionized water revealed spherical homogenous micelles <200 nm in diameter, in agreement with the particle sizing data (Figure 3C). The nanometer dimensions of the particles may be attributable to the fact that P188 and Labrasol self-assemble into micelles at low critical micellar concentrations (CMCs), trapping amphiphilic PMX/DCK molecules [37]. Furthermore, P188 imparts electrostatic and steric stability by increasing packing density and reducing interfacial tension. P188 has been approved as an aid to oral drug delivery and is widely used as a dispersing, emulsifying, and solubilizing agent [38,39]. Additionally, inclusion of a poloxamer is well known to control droplet size in aqueous medium [40]. Labrasol exhibits a high tolerance and promotes absorption through tight junctions in the absence of intestinal membrane damage [41,42]. A previous study confirmed that a molecular dispersion of PMX and DCK exists as an amorphous inclusion complex, enhancing the intestinal membrane permeability of PMX [29]. Thus, incorporation of Labrasol and P188 into our formulation may enhance micellar stability, control particle size, and facilitate intestinal absorption of PMX/DCK by allowing the material to accumulate to high levels on the intestinal surface.

### 3.4. In Vitro Artificial Intestinal Membrane and Caco-2 Cell Permeability

The ability of PMX to permeate an artificial intestinal membrane after ion-pairing complex formation and preparation of PMX/DCK-OP was evaluated. The *P_e_* of PMX after ionic complex formation with DCK was significantly increased, by 5.84-fold, compared with free PMX. This result demonstrated the role of DCK in enhancing passive absorption through a phospholipid layer, which might be due to the increased lipophilicity of PMX complex formation [29]. Moreover, the formulation of PMX/DCK-OP resulted in 255% and 1.976% greater permeability through the artificial membrane than those of PMX/DCK and PMX, respectively (Table 1). The increased permeability may have been due to the use of P188 and Labrasol in the preparation of the oral powder formulation. Using these agents may have increased the partitioning of PMX/DCK into the lipophilic membrane, and disrupted the structural integrity of the lipid bilayer [43]. Furthermore, a similar trend was observed in permeability studies across a Caco-2 monolayer after the ionic complex formation of PMX and DCK, and formulation of PMX/DCK-OP. The *P_app_* of PMX after ionic complex formation with DCK was 3.35-fold higher than that of free PMX, which was attributable to the specific interaction of PMX/DCK with apical sodium-dependent bile acid transporter (ASBT). Moreover, the enhanced paracellular permeability of PMX/DCK might be due to the bile acid-induced phosphorylation of the epidermal growth factor receptor in the Caco-2 monolayer and rearrangement of occludin at the tight junction level [44]. Further, the *P_app_* of PMX/DCK-OP increased significantly, by 3.16- and 10.6-fold of the values for PMX/DCK and PMX, respectively (Table 1). This may reflect micelle formation by PMX/DCK in the presence of Labrasol and P188, enhancing drug absorption via macropinocytosis and clathrin-and caveolae-mediated endocytosis [45,46]. Moreover, the enhanced permeability of the Caco-2 cell monolayer to the powder formulation may indicate Labrasol-induced inhibition of glucuronidation. This inhibition opens junctions via interactions with filamentous actin and the zonula occludens-1 protein, and inhibits secretory transport from enterocytes [47,48,49,50]. Also, Labrasol and P188 may enhance permeation by altering intestinal membrane lipid fluidity and (reversibly) perturbing the epithelial plasma membrane [43,50]. Therefore, the higher *P_app_* of PMX/DCK-OP may be attributable to synergistic activity of DCK, Labrasol, and P188. However, further studies using Labrasol, P188, DCK, and PMX/DCK-OP at higher concentrations are required to ensure that the materials are not toxic to the intestinal membrane.

### 3.5. Pharmacokinetic Property of PMX/DCK-OP in Mice

**Figure 4:**
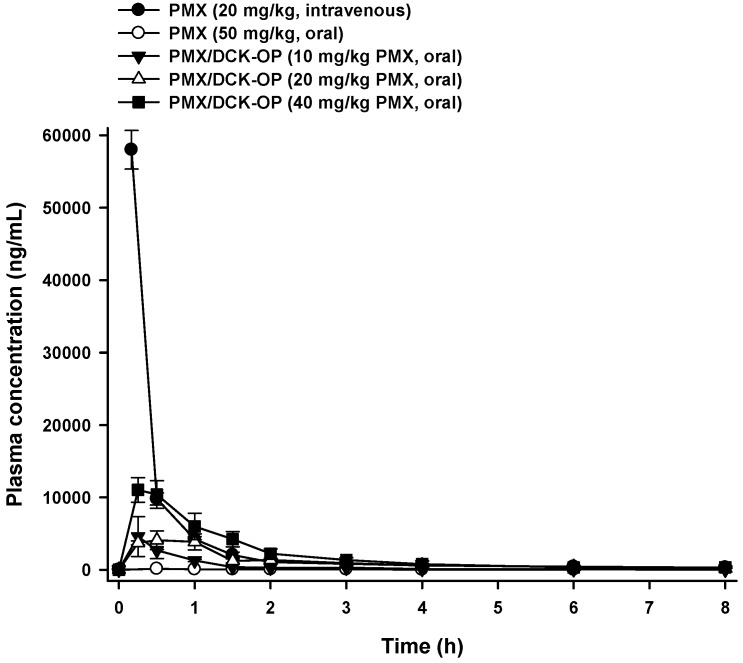
Venous plasma concentration-time profiles of PMX after single intravenous administration of PMX (20 mg/kg) and oral administration of PMX (50 mg/kg), PMX/DCK-OP (10 mg/kg PMX), PMX/DCK-OP (20 mg/kg PMX), or PMX/DCK-OP (40 mg/kg PMX) in mice. Each value represents the mean ± SEM (*n* = 4 for each group).

The mean plasma concentration-time profiles and the pharmacokinetic parameters of PMX after oral administration of an aqueous solution of PMX or different doses of PMX/DCK-OP in mice are presented in Figure 4 and Table 2, respectively. The maximum plasma concentration (C_max_) attained after oral administration of PMX/DCK-OP (40 mg/kg PMX) was 11,032 ± 1693 ng/mL, which was 73.1-fold higher than that of oral PMX (50 mg/kg). Moreover, the C_max_ obtained after oral administration of PMX/DCK-OP (20 mg/kg PMX) was almost similar to that of PMX/DCK-OP (10 mg/kg PMX), which represents dependency of C_max_ on the dose. The area under the plasma concentration-time curve (AUC) after oral administration of PMX/DCK-OP (40 mg/kg PMX) was 53.7-fold greater than that of the oral PMX (50 mg/kg) curve. Moreover, the AUC_last_ values of the PMX/DCK-OP curves were dose- proportional over a drug range of 10–40 mg/kg; the AUC_last_ values were 3622 ± 1001, 8874 ± 1871, and 16,973 ± 3407 ng·h/mL for PMX/DCK-OP equivalents of 10, 20, and 40 mg/kg PMX, respectively. Also, the oral bioavailability of PMX/DCK-OP (40 mg/kg PMX) was 22.8 ± 4.57%, thus 67.1-fold greater than that of oral PMX (50 mg/kg). This significant increase in oral bioavailability might be due to the DCK-induced increase in lipophilicity of PMX, disruption of the intestinal barrier via occludin dephosphorylation, selective interaction of PMX/DCK with the bile acid transporters on the intestinal membrane, as well as Labrasol- and P188-induced alteration of intestinal membrane fluidity. Furthermore, no significant difference in oral bioavailability was observed for PMX/DCK-OP at all three doses administered. In addition, the time to reach the maximum concentration (T_max_) for PMX/DCK-OP (20 mg/kg PMX) was 0.41 ± 0.08 h, which was slightly higher than for PMX/DCK-OP (10 mg/kg PMX) and PMX/DCK-OP (40 mg/kg PMX). Therefore, to confirm the appropriate dose, we further studied the anti-angiogenic and antitumor effects using various doses of PMX/DCK-OP.

### 3.6. In Vivo Anti-Angiogenic Effect of PMX/DCK-OP

**Figure 5:**
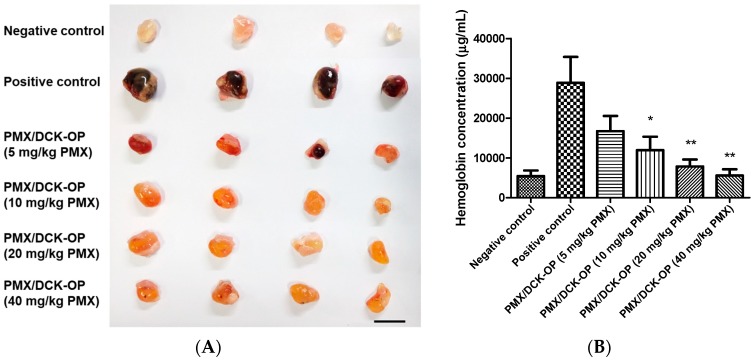
In vivo anti-angiogenic effect of PMX/DCK-OP. Angiogenesis was induced in a subcutaneously implanted Matrigel plug by vascular endothelial growth factor 165 (VEGF_165_) and basic fibroblast growth factor (bFGF) in C57BL/6 mice. The negative control did not contain any growth factors. The negative and positive groups received saline while treatment groups received 5 mg/kg, 10 mg/kg, 20 mg/kg or 40 mg/kg of PMX/DCK-OP based on PMX everyday by the oral route. (**A**) Representative images of extracted Matrigel plugs from C57BL/6 mice at day 7. (**B**) Quantification of hemoglobin content from Matrigel plugs (*n* = 5). Bar means 10 mm. Data are expressed as mean ± SEM. * *p* < 0.05, ** *p* < 0.01 compared to the positive control.

The above in vitro experiments demonstrated that PMX treatments exerted an anti-angiogenic effect. Therefore, we continued by evaluating the anti-angiogenic effect of PMX/DCK-OP. The direct assessment of angiogenesis inhibition in vivo by using implanted Matrigel pellets provided evidence for the anti-angiogenic activity of PMX/DCK-OP. As shown in Figure 5A, Matrigel alone did not induce any angiogenesis within the pellet (negative control) due to the absence of additional growth factors. In contrast, angiogenesis was noticeably induced by adding VEGF_165_ and bFGF (positive control). Despite the relatively short period of treatment (7 days), the metronomic dose of PMX/DCK-OP significantly inhibited VEGF_165_ and bFGF-induced neovascularization in Matrigel implanted mice in a dose-dependent manner by reducing the hemoglobin content by 42.5%, 59.1%, 73.4%, and 81.3% using PMX/DCK-OP (5 mg/kg PMX), PMX/DCK-OP (10 mg/kg PMX) (*p* < 0.05), PMX/DCK-OP (20 mg/kg PMX) (*p* < 0.01), and PMX/DCK-OP (40 mg/kg PMX) (*p* < 0.01), respectively, compared with the positive control group (Figure 5B). The decrease in hemoglobin content indicates a substantial decrease in microvessel formation within the Matrigel plugs due to drug treatment. Thus, this study demonstrates that PMX/DCK-OP successfully permeated the intestinal membrane and inhibited the proliferation and migration of endothelial cells to the Matrigel plug, thereby effectively leading to the inhibition of angiogenesis.

### 3.7. In Vivo Tumor Growth Inhibition Effect of PMX/DCK-OP

**Figure 6:**
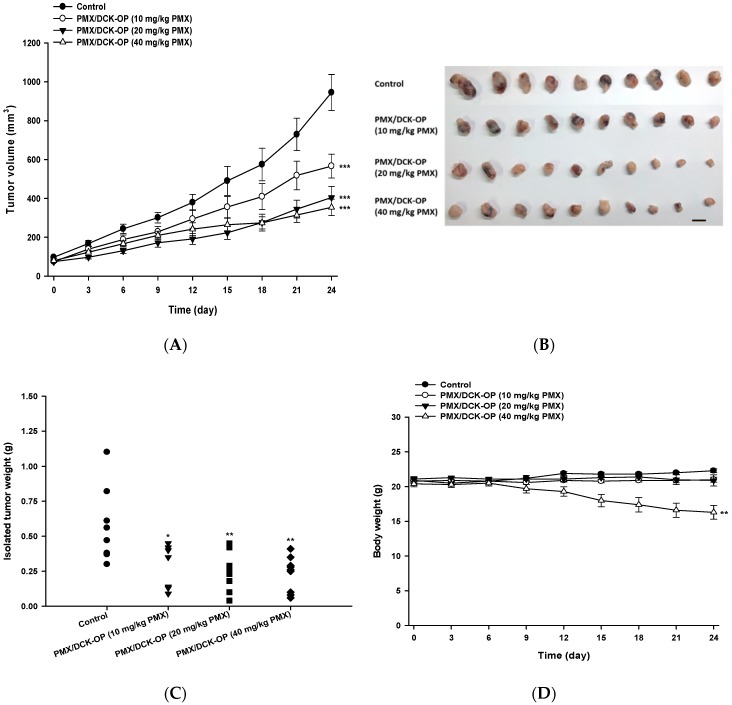
In vivo tumor growth inhibitory effects of PMX/DCK-OP in A549 xenografts. After tumor volume reached 50–70 mm^3^, the mice were randomized (*n* = 10 mice per group) into (1) Control group, receiving saline, (2) the PMX/DCK-OP (10 mg/kg PMX) group, receiving once daily administration of 10 mg/kg PMX/DCK-OP based on PMX, (3) the PMX/DCK-OP (20 mg/kg PMX) group, receiving once daily administration of 20 mg/kg PMX/DCK-OP based on PMX, and (4) the PMX/DCK-OP (40 mg/kg) group, receiving once daily administration of 40 mg/kg PMX/DCK-OP based on PMX. (**A**) Tumor volume of different groups. (**B**) Image of isolated tumors from each group on day 24. (**C**) Tumor weight of mice on day 24. (**D**) Body weight graph of different groups. Bar means 10 mm. Data are expressed as mean ± SEM. * *p* < 0.05, ** *p* < 0.01, *** *p* < 0.001 compared to the control group.

To define an appropriate oral dose of PMX/DCK-OP, the tumor inhibitory effect thereof was assessed using the A549 xenograft model. After 24 days of treatment, PMX/DCK-OP exhibited a dose-dependent tumor inhibitory effect; tumor volumes fell by 40.1, 57.2, and 64.2% when PMX/DCK-OP was given at 10, 20, and 40 mg/kg PMX, respectively, compared to the control group (*p* < 0.001) (Figure 6A). After treatment, all tumors were excised and weighed (Figure 6B). The tumor weight graph well-reflected the tumor volume results; dose-dependent inhibition of tumor volume was apparent. PMX/DCK-OP at 40 mg/kg PMX maximally reduced tumor weight (by 59.1%) compared to the control (*p* < 0.001) (Figure 6C). However, PMX/DCK-OP at that level was somewhat toxic, as indicated by a gradual decrease in body weight of 20.2%; the other treatments were not associated with any notable decrease in body weight (Figure 6D). The study ran for 24 days; we closely observed mouse physical appearance and body weight. All mice survived despite the decrease in body weight of the PMX/DCK-OP (40 mg/kg PMX) group. Thus, we selected PMX/DCK-OP (20 mg/kg PMX) as an appropriate dose for further study; this dose was as effective as PMX/DCK-OP (40 mg/kg PMX) in terms of both anti-angiogenesis and antitumor activities, and did not exhibit any toxic effect. However, further toxicity studies are needed; these should involve repeated oral administration of PMX/DCK-OP at various doses.

### 3.8. In Vivo Anti-Angiogenic Effect of Oral Metronomic Chemotherapy of PMX/DCK-OP

**Figure 7:**
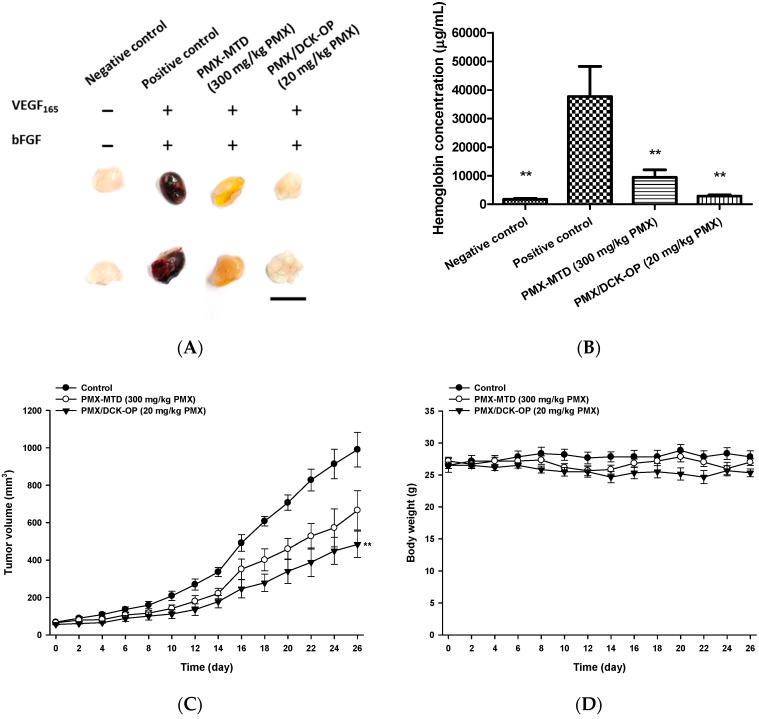
Anti-angiogenic and anti-cancer effect of MCT vs. MTD treatment of PMX. (**A**) Image of Matrigel plugs extracted from C57BL/6 mice inoculated with Matrigel with or without VEGF_165_ and bFGF and receiving saline in the negative and positive control groups, intraperitoneal injection of PMX 300 mg/kg/week as MTD, and oral administration of PMX/DCK-OP 20 mg/kg/day (based on PMX) as MCT. (**B**) Quantification of hemoglobin content from Matrigel (*n* = 5 per group). (**C**) Tumor growth profiles of A549 xenografted mice receiving saline as a control, intraperitoneal injection of PMX 300 mg/kg/week as PMX-MTD, and oral administration of PMX/DCK-OP 20 mg/kg/day (based on PMX) (*n* = 6 per group). (**D**) Body weight profiles. Bar means 10 mm. Data are expressed as mean ± SEM. ** *p* < 0.01 compared to the control group.

After selecting PMX/DCK-OP (20 mg/kg) as an appropriate once-daily oral dose for the metronomic administration of PMX, we compared the anti-angiogenic effects of the metronomic dose with conventional chemotherapy of PMX. Here, once-weekly intraperitoneal administration of 300 mg/kg PMX was used as the MTD dose (PMX-MTD), which is 2.4-times higher than the total of the metronomic doses [51]. First, a Matrigel plug assay was performed as discussed above on the following groups: PMX-MTD (300 mg/kg PMX) and PMX/DCK-OP (20 mg/kg PMX) along with negative and positive controls. A lower level of blood perfusion into the plug was observed in the metronomic group when compared to the positive control and PMX-MTD groups (Figure 7A). Both the treatment groups significantly inhibited blood perfusion in the Matrigel plug with a 75.1% and 92.4% reduction in hemoglobin content observed in the PMX-MTD and PMX/DCK-OP (20 mg/kg PMX) groups, respectively, as compared with the positive control (*p* < 0.01) (Figure 7B). Thus, PMX/DCK-OP (20 mg/kg PMX) significantly inhibited the progression of angiogenesis in the plug.

Next, we compared the anti-cancer effects of MTD and metronomic PMX in A549-xenografted mice. Compared to the control group, the tumor growth rate of lung cancer xenografts was significantly lower in the PMX/DCK-OP (20 mg/kg PMX) group, whereas the PMX-MTD (300 mg/kg PMX) group showed a non-significant reduction in tumor volume. Twenty-six days after treatment commenced, the anti-cancer effect on tumor growth was significantly higher in the PMX/DCK-OP (20 mg/kg PMX) group compared to the control (*p* < 0.01) (51.1% suppression of tumor volume). PMX-MTD (300 mg/kg PMX) reduced tumor volume by only 32.8% (Figure 7C) compared to the control. All treatments were non-toxic (body weight did not fall) (Figure 7D) and no mouse died during the 26 days.

**Figure 8:**
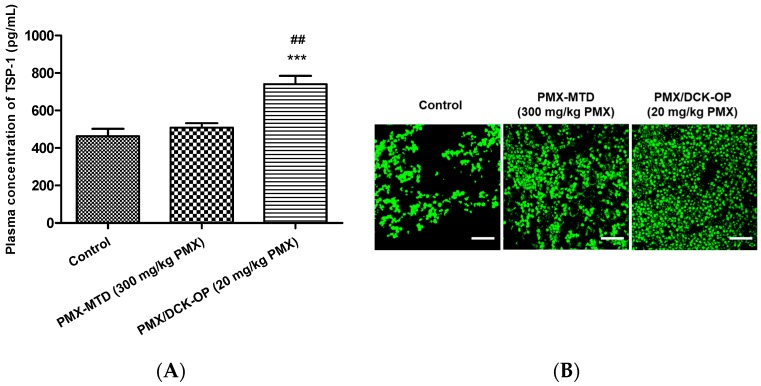

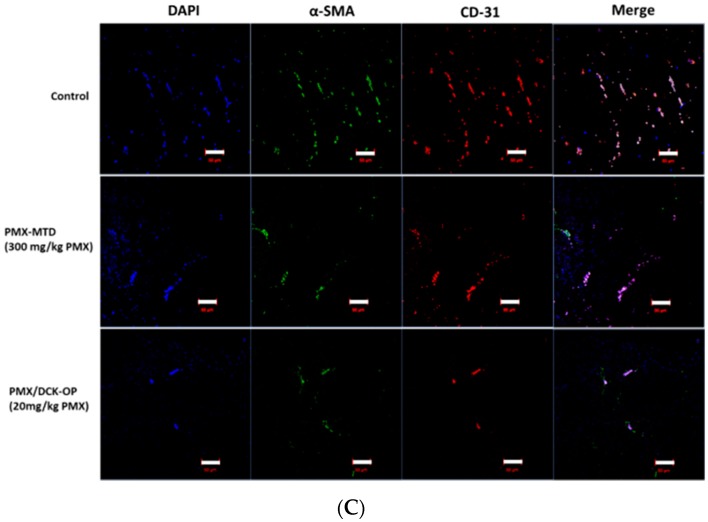
Anti-angiogenic effect of oral metronomic PMX/DCK-OP. (**A**) Quantification of circulating thrombospondin-1 (TSP-1) levels by ELISA in blood plasma withdrawn on the last day of the experiment from mice with A549 xenografts receiving saline (control), intraperitoneally injected PMX at 300 mg/kg/week (MTD), and oral PMX/DCK-OP (20 mg/kg/day of PMX). Data are expressed as means ± SEMs (*n* = 6 per group). *** *p* < 0.001 compared to the control. ^##^ *p* < 0.01 compared to the PMX-MTD (300 mg/kg PMX) group. (**B**) Immunofluorescence analysis of TSP-1 release from tumor tissues isolated on the last day of the experiment from A549 xenografts (*n* = 3). Magnification 800×. Bar: 20 µm. (**C**) Immunofluorescence analysis of blood vessel formation in tumor tissues isolated on the last day of the experiment from A549 xenografts (*n* = 3). The tissues were treated with DAPI (blue), an anti-CD-31 antibody (red), and alpha SMA (green); to stain the nucleus, blood vessels, and pericytes, respectively. Magnification 200×. Bar: 50 µm.

The TSP-1 level indicates the extent of anti-angiogenesis action during metronomic therapy. Several studies have shown that TSP-1 inhibits angiogenesis during metronomic treatment [11,35]. The effect of MCT on the levels of angiogenesis-related factors in the peripheral blood of lung cancer-bearing mice was assessed at the end of treatment. An ELISA revealed that the circulating TSP-1 level in the control group was 463 ± 40.0 pg/mL. The TSP-1 levels in the PMX-MTD (300 mg/kg PMX) and PMX/DCK-OP (20 mg/kg PMX) groups were 509 ± 24.2 and 740 ± 45.3 pg/mL (*p* < 0.001), respectively (Figure 8A). The TSP-1 level of PMX/DCK-OP (20 mg/kg PMX)-treated mice was 1.45-fold greater than that of the PMX-MTD (300 mg/kg PMX) group (*p* < 0.01). Thus, oral MCT of the PMX/DCK-OP (20 mg/kg PMX) group increased the peripheral TSP-1 level. However, conventional PMX-MTD (300 mg/kg PMX) chemotherapy did not increase the TSP-1 level compared to that of the control group, indicating that MTD did not reduce the mobilization or viability of the circulating endothelial precursor cells (CEPs) that contribute to tumor angiogenesis [9]. Tumor growth suppression by PMX-MTD (300 mg/kg PMX) may be attributable to the cytotoxic effect of an antifolate agent, thus not to an anti-angiogenic effect secondarily mediated by TSP-1. On the other hand, metronomic treatment effectively increased the circulating TSP-1 level compared to that of a control group [9]. We next assessed the effect of an endogenous inhibitor of angiogenesis in the tumor microenvironment induced by MCT. To this end, we measured TSP-1 levels in the tumor tissue of lung cancer xenografts. Immunofluorescence staining of tumor sections confirmed that the TSP-1 level was higher in the PMX/DCK-OP (20 mg/kg PMX) group compared to the PMX-MTD (300 mg/kg PMX) and control groups (Figure 8B). Also, tumor sections from PMX/DCK-OP (20 mg/kg PMX) mice showed reduced microvessel density, i.e., less CD-31 staining, compared to the PMX-MTD (300 mg/kg PMX) and control groups, in which tumor vessel density was high. Fewer blood vessels were observed in the metronomic treatment groups (Figure 8C). Similar results were reported by Bocci et al.; hematoxylin-and-eosin-stained tissues (revealing CD-31) from HT-29 tumor-bearing mice exhibited somewhat fewer microvessels (compared to control) in MTD CPT-11-treated tumors, but a dramatic decrease in microvessel numbers in metronomic CPT-11-treated tumors [35]. Thus, oral MCT of PMX/DCK-OP (20 mg/kg PMX) enhanced release of the angiogenesis inhibitor TSP-1 and thus decreased blood vessel formation in the tumor tissue of lung cancer xenografts.

Therefore, the anti-cancer and anti-angiogenic effects of oral MCT were greater in the PMX/DCK-OP group compared with PMX-MTD in a human lung cancer xenograft model.

## 4. Conclusions

In the present study, we developed a new oral formulation of PMX with DCK via ion-pairing complex formation at a 1:1 molar ratio, along with Labrasol and P188 as dispersants (PMX/DCK-OP). The apparent permeability of PMX through a Caco-2 cell monolayer was enhanced 10.6-fold by the oral powder formulation compared with free PMX. Moreover, the oral bioavailability of PMX/DCK-OP (equivalent to 40 mg/kg PMX) in mice was significantly higher compared with that of oral PMX. The daily oral administration of PMX/DCK-OP exhibited a promising anti-angiogenic effect, as observed by decreased hemoglobin content in a Matrigel plug assay. In addition, PMX/DCK-OP exerted significant anti-cancer effects on human lung cancer xenografts. Furthermore, the metronomic administration of PMX/DCK-OP had potential anti-angiogenic and anti-cancer effects compared with an MTD dose of PMX. We believe that this new formulation could be used in metronomic maintenance therapy for patients requiring once-daily administration of chemotherapy. Given its route of administration, patients can very conveniently self-administer at home. This formulation can be beneficial, especially for NSCLC patients requiring maintenance therapy with PMX.

## Figures and Tables

**Table 1:** Effective and apparent permeability of PMX, PMX/DCK, and PMX/DCK-OP.

Test Materials	Effective Permeability (*P_e_*, × 10^−6^, cm/s)	Apparent Permeability (*P_app_*, × 10^−6^, cm/s)
PMX	1.52 ± 0.25	1.78 ± 0.12
PMX/DCK	8.88 ± 0.54 ***^,###^	5.97 ± 1.38 ***^,###^
PMX/DCK-OP	31.6 ± 2.22 ***^,###^	18.9 ± 3.60 ***^,###^

Values are expressed as mean ± standard deviation (*n* = 6). *** *p* < 0.001 compared with PMX; ^###^ *p* < 0.001 compared with PMX/DCK.

**Table 2:** Pharmacokinetic parameters of PMX in mice after intravenous injection of PMX or oral administration of PMX or PMX/DCK-OP with various dose of PMX.

Test Materials	PMX (20 mg/kg, IV)	PMX (50 mg/kg, Oral)	PMX/DCK-OP (10 mg/kg PMX, Oral)	PMX/DCK-OP (20 mg/kg PMX, Oral)	PMX/DCK-OP (40 mg/kg PMX, Oral)
Administration	Intravenous	Oral	Oral	Oral	Oral
Dose of PMX (mg/kg)	20	50	10	20	40
T_max_ (h)	0.17 ± 0.00	0.44 ± 0.05	0.33 ± 0.08	0.41 ± 0.08	0.25 ± 0.00
T_1/2_ (h)	2.57 ± 0.57	2.62 ± 0.67	3.08 ± 2.18	2.48 ± 0.27	1.57 ± 0.42
C_max_ (ng/mL)	58,000 ± 2669	151 ± 5.77	4653 ± 2728	4558 ± 913	11,032 ± 1693
AUC_last_ (ng·h/mL)	37,294 ± 928	316 ± 31.6	3622 ± 1001	8874 ± 1871	16,973 ± 3407
AUC_inf_ (ng·h/mL)	38,521 ± 1.129	443 ± 62.2	4755 ± 674	9604 ± 1868	17,944 ± 3346
Bioavailability (%)	100	0.34 ± 0.01	19.4 ± 5.37	23.8 ± 5.02	22.8 ± 4.57

T_max_: time to reach maximum plasma concentration; T_1/2_: half-life of plasma concentration; C_max_: maximum plasma concentration; AUC_last_: area under the plasma concentration-time curve from zero to the time of the last measurable plasma concentration; AUC_inf_: area under the plasma concentration-time curve from zero to infinity; bioavailability (%): (AUC_last, oral_/Dose_PMX, oral_)/(AUC_last, intravenous_/Dose_PMX, intravenous_) × 100. Values are expressed as mean ± SEM (*n* = 4).
